# Innovation in Times of Uncertainty: Clinical Trials in Nursing Homes During SARs-CoV-2

**DOI:** 10.1093/geroni/igab046.391

**Published:** 2021-12-17

**Authors:** Joan Carpenter

**Affiliations:** University of Maryland School of Nursing, Baltimore, Maryland, United States

## Abstract

In March 2020 the Centers for Medicare & Medicaid Services (CMS) announced restrictions on visitors and nonessential personnel in nursing homes to protect residents and facilities from SARS-CoV-2 (severe acute respiratory syndrome coronavirus 2) outbreaks. At the time, these measures were “temporary” but they continued well into 2021 resulting in a prolonged pause on in-person study activities in a palliative care clinical trial in 12 nursing homes. This session will address the impact of this pause and decisions made to overcome the potential failure of the trial. Of utmost importance was respecting nursing homes rapidly changing context, continued communication with the site leadership, transitioning to phone and video-conference study activities, and designing a retrospective study using existing data to answer a different but similar research question. As clinical researchers move forward implementing trials and complex interventions in nursing homes, we must use the lessons learned to design flexible trial protocols.

